# Substrate stiffness effect and chromosome missegregation in hIPS cells

**DOI:** 10.1186/s12952-015-0042-8

**Published:** 2015-12-18

**Authors:** Suehelay Acevedo-Acevedo, Wendy C. Crone

**Affiliations:** Department of Biomedical Engineering, University of Wisconsin-Madison, Madison, WI 53706 USA; Department of Engineering Physics, University of Wisconsin-Madison, Madison, WI 53706 USA

**Keywords:** Induced pluripotent stem cells, Chromosome missegregation, Mitosis, Substrate stiffness

## Abstract

**Background:**

Ensuring genetic stability in pluripotent stem cell (PSC) cultures is essential for the development of successful cell therapies. Although most instances lead to failed experiments and go unreported in the literature, many laboratories have found the emergence of genetic abnormalities in PSCs when cultured in vitro for prolonged amounts of time. These cells are primarily cultured in non-physiological stiff substrates like tissue culture polystyrene (TCPS) which raises the possibility that the cause of these abnormalities may be influenced by substrate mechanics.

**Findings:**

In order to investigate this, human PSCs were grown on substrates of varying stiffness such as a range of polyacrylamide formulations, TCPS, and borosilicate glass coverslips. These substrates allowed for the testing of a stiffness range from 5kPa to 64GPa. Two human induced PSC (iPSC) lines were analyzed in this study: 19-9-11 iPSCs and 19.7 clone F iPSCs. Centrosome and DNA staining revealed that 19-9-11 iPSCs range from 1–8.5 % abnormal mitoses under the different culture conditions. A range of 4.4–8.1 % abnormal mitoses was found for 19.7 clone F iPSCs.

**Conclusions:**

Abnormal cell division was not biased towards one particular substrate. It was confirmed by Analysis of Variance (ANOVA) and Tukey’s Honest Significant Difference test that there was no statistically significant difference between passage numbers, cell lines, or substrates.

**Electronic supplementary material:**

The online version of this article (doi:10.1186/s12952-015-0042-8) contains supplementary material, which is available to authorized users.

## Background

Stem cell research aims to grow human cells in vitro for transplantation and carry out drug and toxicity screenings on relevant human cell and tissue models. In order for these goals to be successful, it is important to conserve the genetic stability of these cells. Several reports assessing the genetic stability of human pluripotent stem cells (PSCs), including both embryonic and induced stem cell lines, when cultured in vitro for prolonged amounts of time have been published in recent years [1–4]. It has been suggested that chromosomal gains, such as trisomies 8, 12, 17 or X, give PSCs a selective advantage in in vitro culture due to the fact that chromosome 12, for example, encodes for many cell-cycle related genes [5].

A recent study done by Holubcová et al. [6] found the frequency of multicentrosomal mitosis (defined as a mitotic cell with more than 2 centrosomes) for human embryonic stem cells to be between 10 and 23 %. Additionally, Gisselsson et al. [7] determined that the chromosome missegregation rate for normal dermal fibroblasts was about 1 missegregation event in around 50 cell divisions.

How the mechanics of the surroundings influence a dividing cell in vitro is beginning to be investigated. For example, Kocgozlu et al. [8] reported that softer substrates hinder epithelial cell division by leading to abnormal morphology in chromosome segregation. Tse et al. [9] found that mechanically confined HeLa cell divisions resulted in an increase in abnormal multi-daughter divisions. Given this evidence in differentiated cell types, it can be speculated that the observed recurrent chromosomal abnormalities may be an artifact of in vitro culture, particularly when taking into account that embryonic stem cells exist in vivo for a short period of time.

A common factor between the abnormal cell lines reported is that they were cultured on stiff substrates like glass (E = 60–64GPa; [10]) and tissue culture polystyrene (TCPS; E = 2.28–3.28 GPa; [11]). For comparison, human tissues such as neural, muscle and collagenous bone fall into a stiffness range of 1–100 kPa. Many studies have investigated the effect of mechanical stimuli on stem cell fates such as self-renewal [12, 13] and differentiation [14, 15].

This study began with the hypothesis that culturing PSCs in non-physiologic stiff culture conditions such as TCPS and glass caused abnormal mitotic spindle formation and/or spindle function due to substrate stiffness, consequently causing chromosome missegregation. To determine the effect of matrix stiffness on PSCs, culture substrates commonly used for in vitro culture such as TCPS and glass were evaluated in addition to a matrix whose stiffness could be readily altered in order to study a biologically relevant range. Polyacrylamide (PA) hydrogels were chosen due to ease of fabrication, tuneability of mechanical properties and amount of studies done using this polymer to assess the effect of stiffness on different cell fates [14, 16–18].

## Materials and methods

### Cell culture

The cell lines used were 19.7 clone F induced pluripotent stem cells (iPSCs) and DF19-9-11 iPSCs acquired from WiCell [19] and approved by the Stem Cell Research Oversight (SCRO) Committee at the University of Wisconsin-Madison. All cell lines were cultured as described previously by Chen et al. [20]. Passage number since derivation for DF19-9-11 iPSCs ranged from passage 34–55 and from passage 51–65 for 19.7 clone F iPSCs.

### Polyacrylamide hydrogel fabrication and functionalization

The hydrogels were fabricated using a method described by Hazeltine et al. [18]. The surfaces of the hydrogels were functionalized with a N-sulfosuccinimidyl-6-[4′-azido-2′-nitrophenylamino] (Sulfo-SANPAH) treatment as described previously by Hazeltine et al. [18, 21] in order to facilitate attachment of a thin surface coating with Matrigel to promote cell adhesion, allow for force transduction between the cell and the substrate, and maintain pluripotency of iPSCs.

### Mechanical testing

Polyacrylamide (PA) samples designated for mechanical characterization were polymerized into a standard tensile test geometry following ASTM standard D638-08 [22]. Stiffness of the specific PA gel formulations used in this research was determined by tensile testing using an Instron 5548 MicroTester, with a 10 N load cell. Using a tensile test method developed in the lab for hydrogel materials [23], the Young’s modulus of the PA formulations were measured and summarized in Table 1.

**Table 1:** Summary of Young’s modulus obtained for different culture substrates

Substrate	Acrylamide Concentration (%)	Bisacrylamide Concentration (%)	Young’s modulus (Pa)
No.1 borosilicate glass coverslips	-	-	60–64 × 10^9^
Tissue Culture Polystyrene	-	-	2.28–3.28 × 10^9^
Polyacrylamide	10 %	0.03 %	5.0 × 10^3^ ± 1.6
	10 %	0.3 %	31.0 × 10^3^ ± 5.7
	10 %	0.6 %	51.7 × 10^3^ ± 6.0
	10 %	1.2 %	59.6 × 10^3^ ± 14.7

### Immunofluorescence

Human iPSCs were fixed using a 4 % paraformaldehyde (Electron Microscopy Sciences), 0.3 % glutaraldehyde (Sigma), 0.1 % Triton X (Sigma) solution in cytoskeletal buffer [24]. Samples were post-fixed with 90 % ice-cold methanol (Sigma) and quenched with 100 mM sodium borohydride (Sigma). Samples were blocked in 50 μg/mL bovine serum albumin (BSA; Sigma), 0.1 % Triton X, 150 mM glycine (Sigma) and goat serum (Sigma) in phosphate buffered saline (PBS) for 30 min. The antibodies used in this study were anti-γ-tubulin (4D11; Thermo Scientific), anti-phosphorylated Histone 3 (pH3; 9H12L10; Life Technologies), anti-Oct4 (H-134; Santa Cruz Biotechnology, Inc.), goat anti-rabbit IgG-H&L (Cy3; Abcam) and goat anti-mouse IgG-H&L (DyLight488; Abcam). Nuclei were stained with ProLong Gold Antifade Reagent with DAPI (4',6-diamidino-2-phenylindole; Life Technologies). Samples were imaged using a Nikon Eclipse T*i* inverted epifluorescence microscope system with a 40x objective for mitotic index quantifications and a 60x oil objective for abnormal mitosis quantification.

### Statistical analysis

ANOVA and Tukey’s Honest Significant Difference test were performed using R software [25].

## Results and discussion

To determine the effect of stiffness on human iPS cell division, karyotypically normal human 19-9-11 iPSCs and abnormal 19.7 clone F iPSCs were cultured on substrates with different Young’s modulus values as summarized in Table 1. Glass and TCPS were used because cells are usually cultured on these substrates during regular cell maintenance or for imaging applications. The mechanical properties of PA hydrogels were altered by varying the bisacrylamide cross-linker concentration from 0.03–1.2 % thus generating a stiffness range of 5–60 kPa.

**Fig. 1:**
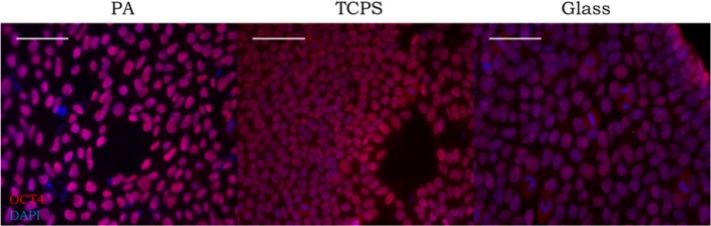
Human iPSCs remain pluripotent on substrates of varied stiffness. Nuclei are stained blue with DAPI and OCT4+ cells are labeled in red. Epifluorescence images of 19-9-11 iPSCs cultured on 31kPA hydrogels (*left panel*), TCPS (*middle panel*) and glass coverslips (*right panel*). Scale bar for TCPS image: 100 μm. Scale bar for PA and glass images: 50 μm

To determine stiffness effects on mitotic spindle morphology and organization, mitotic figures were examined for 5 consecutive passages and at passage 10 on glass and TCPS. Cells growing on PA were analyzed after 4 days in culture. Due to low density of cells that would remain attached until passaging (around 4 days), quantification of abnormalities for cells cultured on PA was not able to be carried out for multiple passages as with glass and TCPS. Cells were still pluripotent on all substrates as confirmed by OCT4 expression (Fig. 1) which has been shown is essential for maintenance of pluripotency in human and mouse PSCs [26, 27]. From immunofluorescence images, the mitotic index was quantified by dividing the number of pH3 positive cells by the total number of cells. Mitotic index quantification for 19-9-11 iPSCs cultured on glass, TCPS and PA (Additional file 1: Figure S1) revealed similar percentages for the different substrates. No statistically significant difference was found for the substrates by ANOVA and Tukey’s Honest Significant Difference test.

**Fig. 2:**
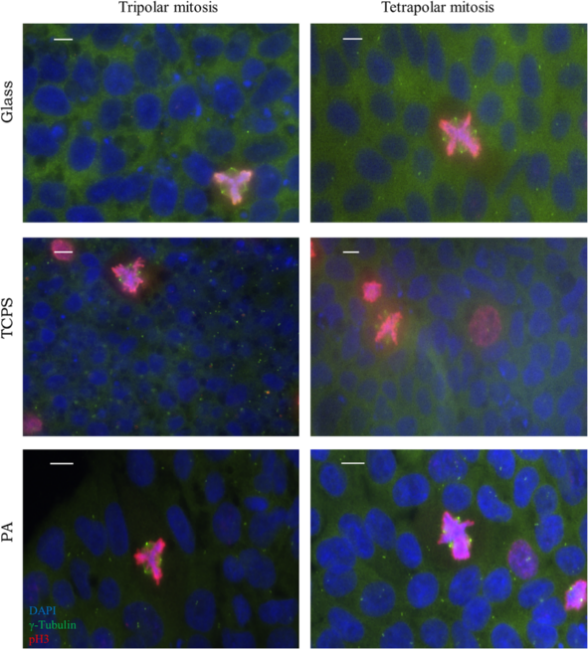
Commonly observed multipolar mitoses in 19-9-11 iPSCs cultured on substrates of varied stiffness. Nuclei are labeled in *blue* (DAPI), γ-tubulin is labeled in *green* while pH3 is labeled in *red*. The rows indicate the substrates human iPSCs were cultured on. Tripolar mitoses are characterized by 3 spindle poles (green γ-tubulin foci). Tetrapolar mitoses are characterized by 4 spindle poles. These types of abnormalities are included in the percentage of abnormal mitoses calculated in Figs. 3 and 4. Scale bars: 10 μm

**Fig. 3:**
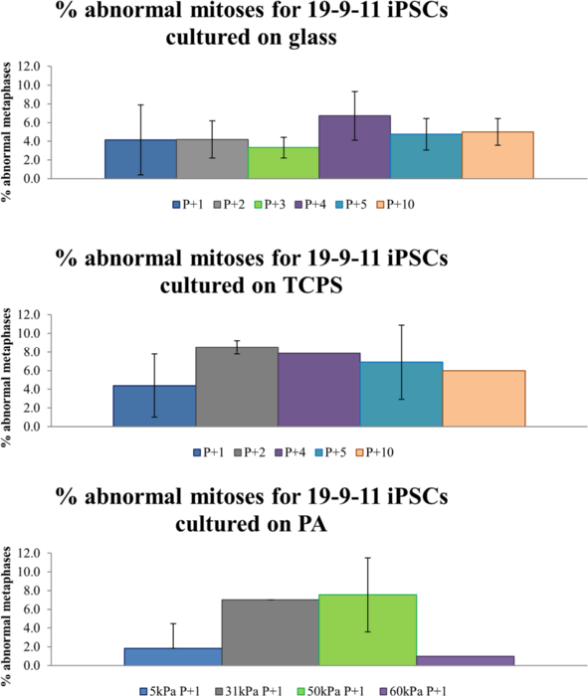
Frequency of abnormal mitoses for 19-9-11 iPSCs cultured on glass, TCPS and PA hydrogels. Bar plot for 19-9-11 iPSCs cultured on glass show percent of abnormal mitoses for 5 consecutive passages and passage 10. Plot for TCPS shows quantification for passages 1, 2, 4, 5 and 10. Passage 3 was unable to be quantified due to contamination. Quantification for PA hydrogels was done only for one passage. n = 26–132 mitoses per condition for 1–5 independent experiments done. Passage number since derivation for this cell line ranged from passage 34–55

**Fig. 4:**
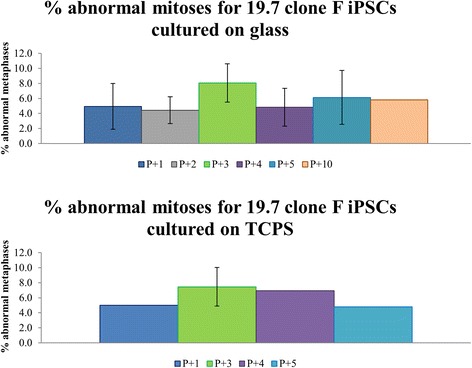
Frequency of abnormal mitoses for 19.7 clone F iPSCs cultured on glass and TCPS. Bar plot for 19.7 clone F iPSCs cultured on glass show percent of abnormal mitoses for 5 consecutive passages and passage 10. Plot for TCPS shows quantification for passages 1, 3, 4 and 5. Passage 2 was unable to be quantified due to contamination. n = 88–112 mitoses per condition for 1–5 independent experiments done. Passage number since derivation for this cell line ranged from passage 51–65

For this study, abnormal mitoses are defined as any prometaphases and metaphases that have 3 or more centrosomes or misaligned chromosomes (chromosomes separated from the rest of the chromosomes located at the metaphase plate). Abnormal mitotic spindles shown in Fig. 2 (Additional file 2: Figure S2 and Additional file 3: Figure S3) represent the different types of abnormalities that were pooled into the percentage of abnormal mitoses quantified in Figs. 3 and 4. Observed mitotic spindles with 3 or 4 centrosomes displaying triangular or square spindle morphology respectively are shown in Fig. 2. Other abnormalities observed include misaligned chromosomes, mitotic spindles with more than 4 centrosomes and potentially inactive centrosomes (Additional file 2: Figure S2 and Additional file 3: Figure S3). Abnormalities in anaphase such as lagging chromosomes and multipolar chromosome segregation during anaphase were also observed but not quantified (Additional file 4: Figure S4).

Summarized in Figs. 3 and 4 are the percentages of abnormal mitoses quantified from γ-tubulin (centrosome marker) and pH3 (mitotic marker) staining. Quantification of abnormal mitoses revealed that these occur at a frequency of up to 8.5 %. For the 19-9-11 cell line, iPSCs cultured on Matrigel-coated glass had a percentage of abnormal mitoses of 3.3 to 6.7 %. A range of 4.4 to 8.5 % abnormal mitoses was found for iPSCs cultured on TCPS. For cells grown on PA gels, 60 kPa PA had the lowest percentage abnormal mitoses with only 1 %. The percentage then increased with 1.9 % for 5 kPa, 7 % for 31 kPa and 7.6 % for 48 kPa hydrogels.

For the 19.7 clone F iPSC line, iPSCs cultured on Matrigel-coated glass coverslips had a percentage of abnormal mitoses of 4.4 to 8.1 %. A range of 4.8 to 7.5 % abnormal mitoses was found for iPSCs cultured on TCPS. Previous G-banding karyotype analysis of this line, supplied by WiCell, revealed that 20 % of the population was abnormal (data not shown).

These results are in discrepancy with the results published by Holubcová and colleagues [6] which report that human embryonic stem cells have a frequency of multicentrosomal mitosis of 10–23 %. A possible source of discrepancy is a cell line-specific susceptibility to centrosome amplification or substrate stiffness influence. Holubcová et al. found that different substrate-extracellular matrix (ECM) coating combinations altered the frequency of multicentrosomal mitosis. When comparing all substrates in the work reported here, the percent of abnormal mitoses did not follow any observable trend or bias with stiffness. Additionally, there were no differences between cell lines or passage number. This was confirmed by ANOVA and Tukey’s Honest Significant Difference test which found no statistically significant difference between the conditions. An explanation for these different results could be that cell-ECM interactions are influencing the frequency of multicentrosomal mitosis instead of cell-substrate interactions. Further studies are required to answer this question.

Alternatively, culture system differences (i.e. ECM coating, media, Rho-associated protein kinase (ROCK) inhibitor) may be the cause. The constant treatment with ROCK inhibitor in this prior study could be responsible since it has been shown that cells treated with ROCK inhibitor exhibit improper mitotic spindle positioning and assembly [28]. A future study of abnormal mitoses for iPSCs cultured under prolonged ROCK inhibition is warranted.

Despite the percentage of abnormal mitoses being lower than expected, the types of abnormalities found were varied. The presence of multipolar mitoses as a result of additional centrosomes may suggest a deregulation of the centrosome duplication cycle or failed cytokinesis particularly for cells that contain a large number of centrosomes such as those shown in Additional file 2: Figures S2A and S2B. Mechanisms that affect centrosome duplication have been mainly studied in the context of cancer and are reviewed in Meraldi et al. [29]. On the other hand, the presence of lagging and misaligned chromosomes may suggest altered microtubule dynamics such as incorrect chromosome-microtubule attachments or changes in motor proteins associated with microtubule positioning. Future studies addressing the roles of centrosome duplication errors or altered microtubule dynamics in the context of human PSC chromosome missegregation could provide insight into the mechanisms behind observed recurrent chromosomal abnormalities in these cells.

## Additional files

Additional file 1: Figure S1. Bar plot showing the mitotic index for 19-9-11 iPSCs cultured on substrates of varied stiffness. n = 197–1,132 total cells counted per condition for 2–3 independent experiments per culture substrate. (TIF 84 kb) Additional file 2: Figure S2. Examples of additional cell division abnormalities seen in 19-9-11 iPSCs on different substrates. Nuclei are labeled in *blue* (DAPI), γ-tubulin is labeled in *green* while pH3 is labeled in *red*. The rows indicate the substrates human iPSCs were cultured on. The left panels show representative images of multipolar mitoses where the centrosomes, indicated by γ-tubulin staining, have different morphology as those seen in Fig. 2. The right panels show misaligned chromosomes which are separated from the chromosomes aligned at the metaphase plate. These types of abnormalities are included in the percentage of abnormal mitoses calculated in Fig. 3. Scale bars: 10 μm. (TIF 4853 kb) Additional file 3: Figure S3. Abnormalities found in 19-9-11 iPSCs not common to all substrates. Nuclei are stained in *blue* (DAPI), γ-tubulin is labeled in *green* while pH3 is labeled in *red*. (A) Abnormal mitosis of an iPSC cultured on glass with 7 centrosomes indicated by bright γ-tubulin stain. (B) Multipolar mitosis of an iPSC cultured on TCPS with 6 centrosomes (C) A tripolar mitosis of an iPSC cultured on 31 kPa PA with a fourth potentially inactive centrosome. These types of abnormalities are included in the percentage of abnormal mitoses calculated in Fig. 3. Scale bars: 10 μm. (TIF 2417 kb) Additional file 4: Figure S4. Examples of abnormal anaphases in 19-9-11 iPSCs cultured on substrates of varied stiffness. Nuclei are stained in *blue* (DAPI), γ-tubulin is labeled in *green* while pH3 is labeled in *red*. Representative images of multipolar anaphases (left panels) and lagging chromosomes (right panels) detected in 19-9-11 iPSCs. Multipolar anaphases are cells that segregate their chromosomes improperly to more than two daughter cells. Lagging chromosomes are chromosomes that segregate separately from the rest of the chromosomes after anaphase onset, usually due to the chromosomes being attached to both spindle poles. The rows indicate the substrates human iPSCs were cultured on. These types of abnormalities are **not** included in the percentage of abnormal mitoses calculated in Fig. 3. Scale bars: 10 μm. (TIF 5065 kb)

## Abbreviations

PSCs: Pluripotent stem cells
iPSCs: Induced pluripotent stem cells
TCPS: Tissue culture polystyrene
ANOVA: Analysis of variance
PA: Polyacrylamide
SCRO: Stem cell research oversight
Sulfo-SANPAH: N-sulfosuccinimidyl-6-[4′-azido-2′-nitrophenylamino]
BSA: Bovine serum albumin
PBS: Phosphate buffered saline
pH3: Phosphorylated histone 3
DAPI: (4', 6-diamidino-2-phenylindole)
ECM: Extracellular matrix
ROCK: Rho-associated protein kinase
