# Supplementary figures and images for: Substrate stiffness effect and chromosome missegregation in hIPS cells
Source: J Negat Results Biomed. 2015 Dec 18;14:22. doi: 10.1186/s12952-015-0042-8 (PMC4683860; doi:10.1186/s12952-015-0042-8)

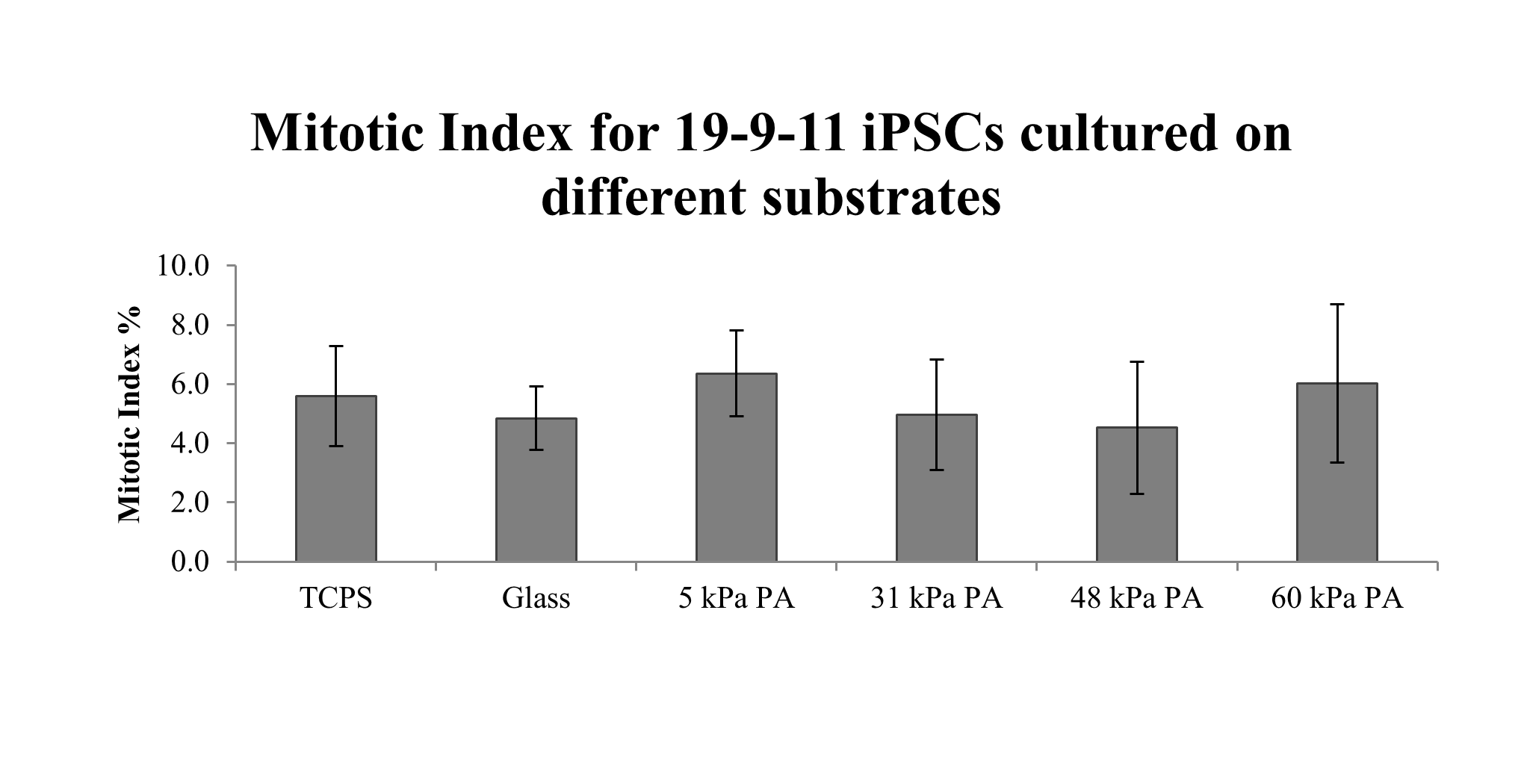

Supplement: Additional file 1: Figure S1. — Bar plot showing the mitotic index for 19-9-11 iPSCs cultured on substrates of varied stiffness. n = 197–1,132 total cells counted per condition for 2–3 independent experiments per culture substrate. (TIF 84 kb) [file 12952_2015_42_MOESM1_ESM.tif]

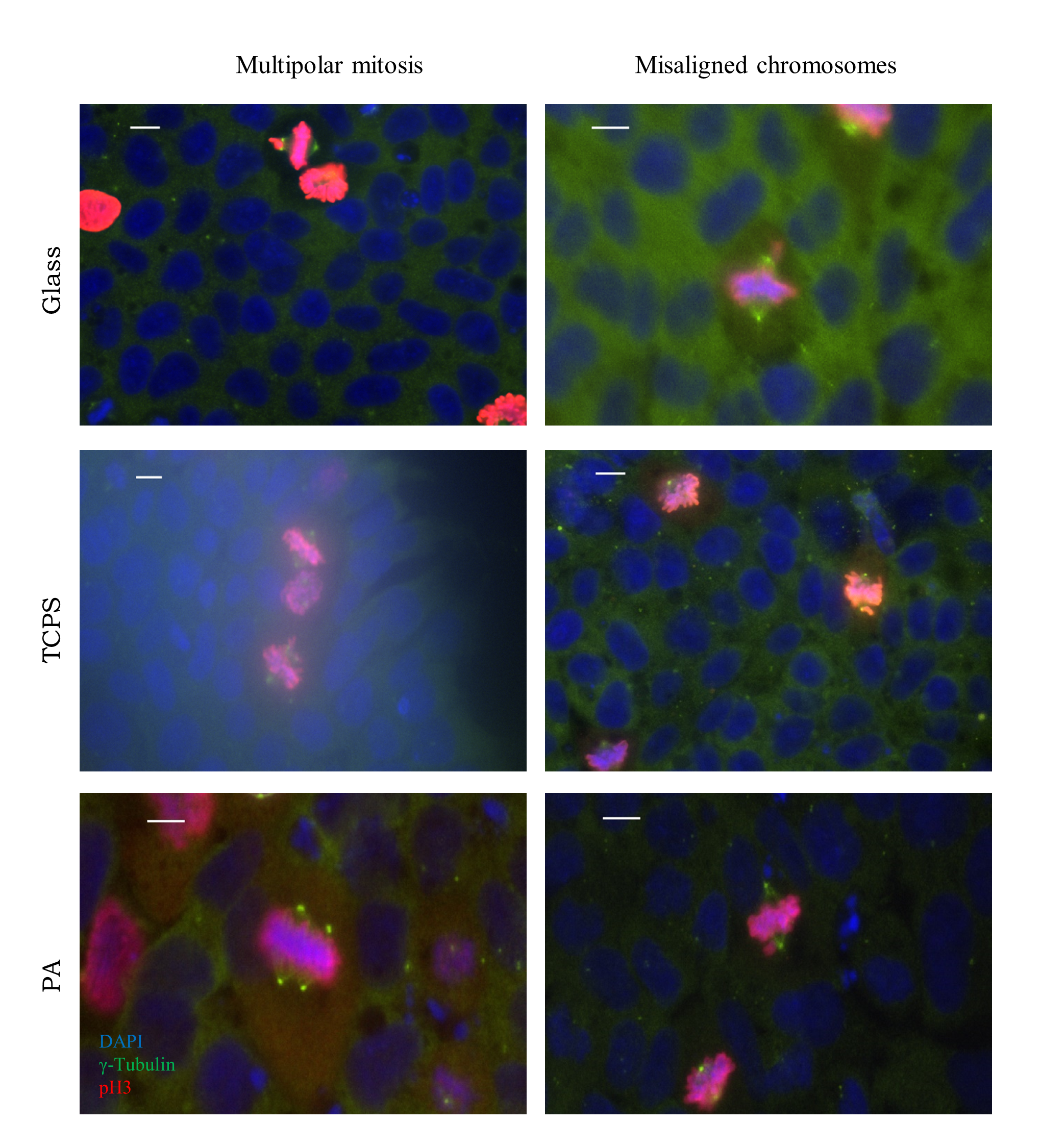

Supplement: Additional file 2: Figure S2. — Examples of additional cell division abnormalities seen in 19-9-11 iPSCs on different substrates. Nuclei are labeled in blue (DAPI), γ-tubulin is labeled in green while pH3 is labeled in red. The rows indicate the substrates human iPSCs were cultured on. The left panels show representative images of multipolar mitoses where the centrosomes, indicated by γ-tubulin staining, have different morphology as those seen in Fig. 2. The right panels show misaligned chromosomes which are separated from the chromosomes aligned at the metaphase plate. These types of abnormalities are included in the percentage of abnormal mitoses calculated in Fig. 3. Scale bars: 10 μm. (TIF 4853 kb) [file 12952_2015_42_MOESM2_ESM.tif]

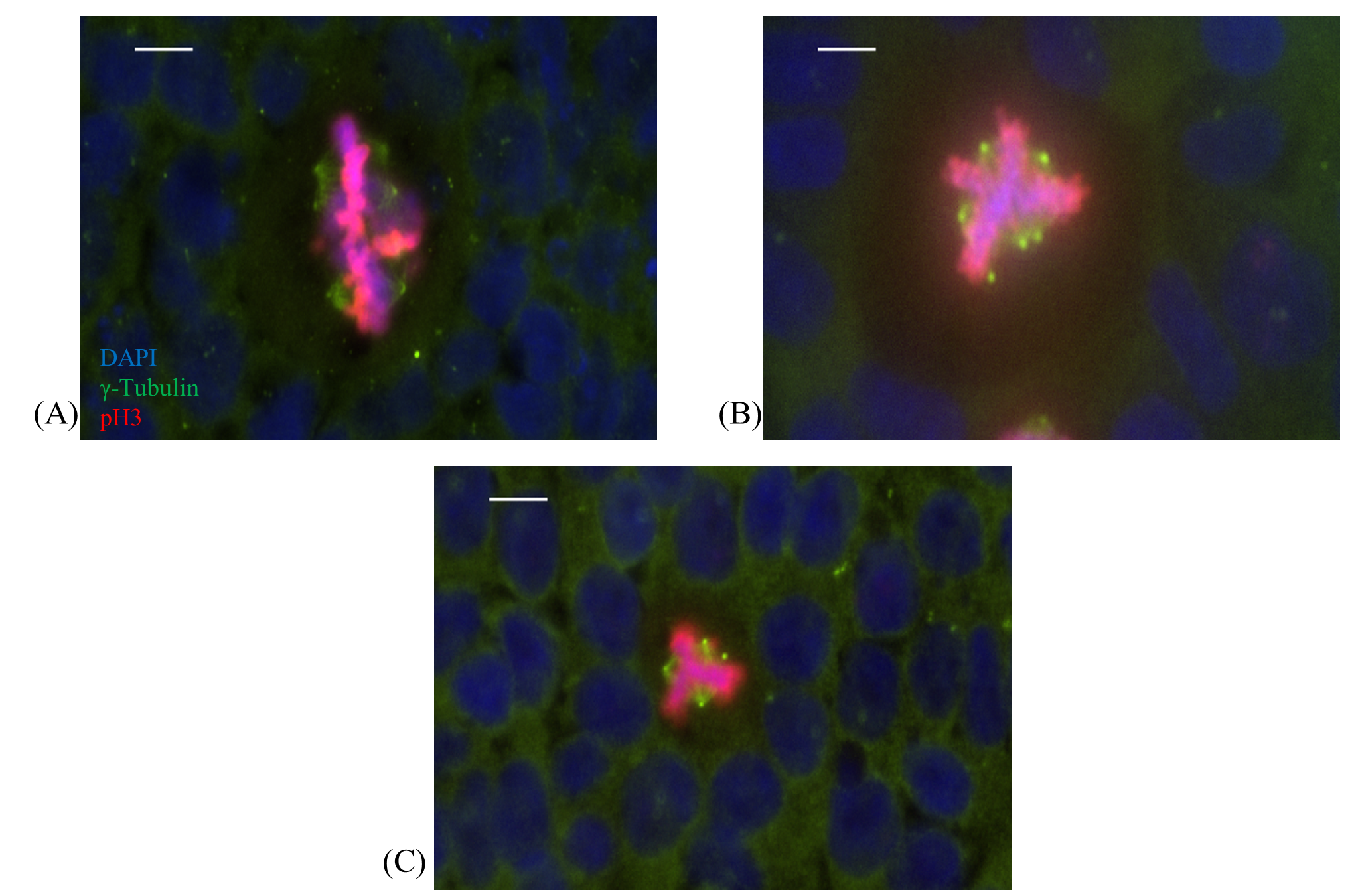

Supplement: Additional file 3: Figure S3. — Abnormalities found in 19-9-11 iPSCs not common to all substrates. Nuclei are stained in blue (DAPI), γ-tubulin is labeled in green while pH3 is labeled in red. (A) Abnormal mitosis of an iPSC cultured on glass with 7 centrosomes indicated by bright γ-tubulin stain. (B) Multipolar mitosis of an iPSC cultured on TCPS with 6 centrosomes (C) A tripolar mitosis of an iPSC cultured on 31 kPa PA with a fourth potentially inactive centrosome. These types of abnormalities are included in the percentage of abnormal mitoses calculated in Fig. 3. Scale bars: 10 μm. (TIF 2417 kb) [file 12952_2015_42_MOESM3_ESM.tif]

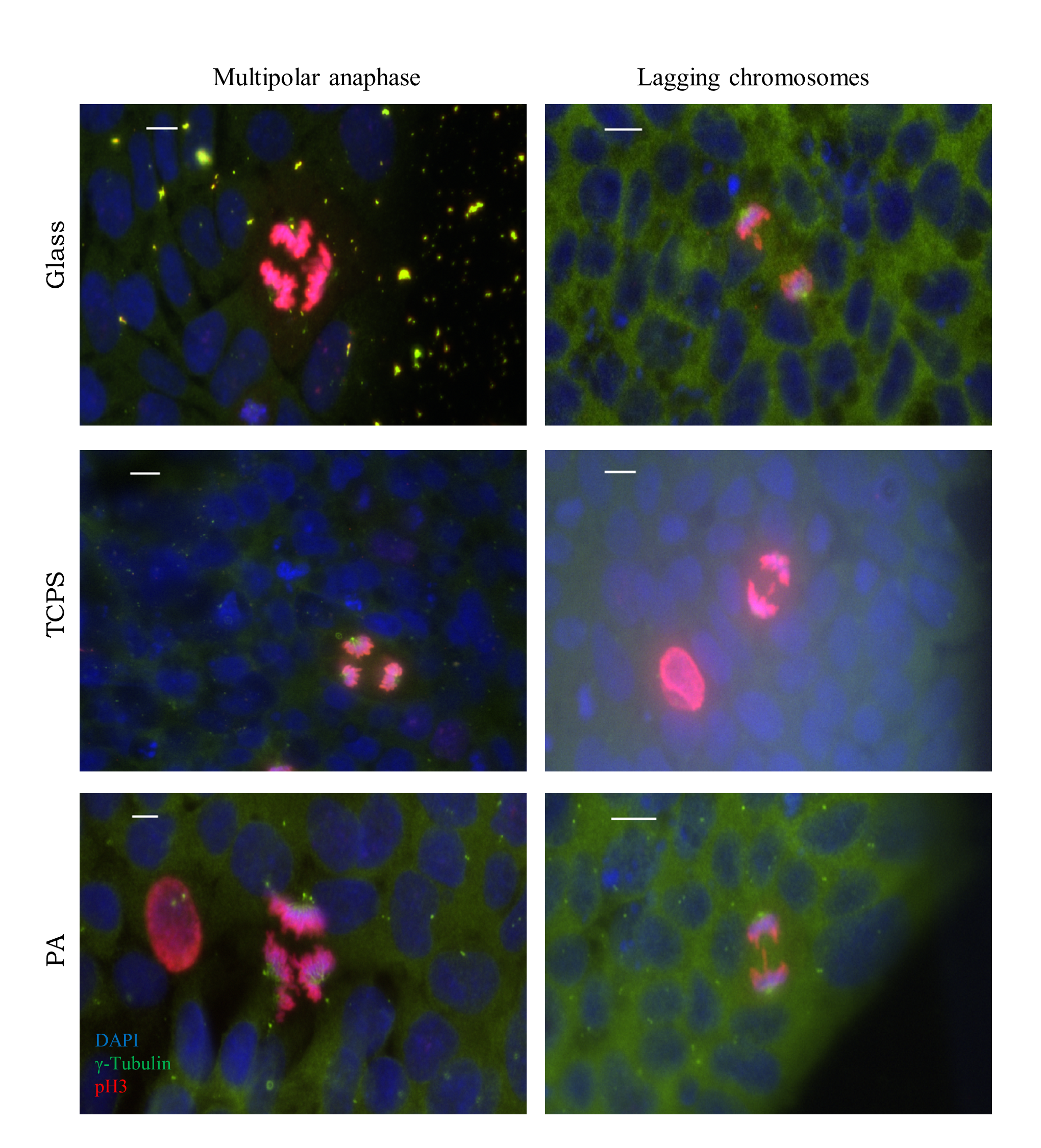

Supplement: Additional file 4: Figure S4. — Examples of abnormal anaphases in 19-9-11 iPSCs cultured on substrates of varied stiffness. Nuclei are stained in blue (DAPI), γ-tubulin is labeled in green while pH3 is labeled in red. Representative images of multipolar anaphases (left panels) and lagging chromosomes (right panels) detected in 19-9-11 iPSCs. Multipolar anaphases are cells that segregate their chromosomes improperly to more than two daughter cells. Lagging chromosomes are chromosomes that segregate separately from the rest of the chromosomes after anaphase onset, usually due to the chromosomes being attached to both spindle poles. The rows indicate the substrates human iPSCs were cultured on. These types of abnormalities are not included in the percentage of abnormal mitoses calculated in Fig. 3. Scale bars: 10 μm. (TIF 5065 kb) [file 12952_2015_42_MOESM4_ESM.tif]
